# The Medical Association of Central New York

**Published:** 1901-10

**Authors:** 


### The Medical Association of Central New York.

THE Medical Association of Central New York held its 34th annual meeting at Buffalo, N.Y., on August 27 and 28, 1901. In point of attendance, quality of papers presented at the sessions and cordiality among the members, this meeting must rank as one of the best ever held by the association. An innovation was made in having two morning sessions, because of the attractions at the Pan-American Exposition, and on this account the meeting was held six weeks ahead of the usual time. This prevented the attendance of many of the older members who generally take their vacation during the month of August, and their faces were missed at the different places of assemblage. Meeting during the exposition season, the members were entertained by a committee of Buffalo physicians, who were generously assisted by several of the exposition concessionnaires, notably among the latter were Mr. Frank C. Bostock, the animal king, who kindly sent Esau, the educated Chimpanzee, to the morning sessions of the second day, where a spirited discussion arose as to his place in the evolution of man. Dr. Coney, of the Infant Incubators, invited the association to a special demonstration in order to study more carefully the many advantages offered by these life-saving inventions. Wenona, of the Indian Congress, consented to appear at the dinner given at the "Streets of Mexico" and showed her skill and expertness with the rifle. Mr. Locke had charge of the dinner and served it in real Mexican style.

On the whole a most enjoyable time was spent on the grounds and the members were full of praise for the hospitality accorded by the Buffalo physicians and their allies. A full report of the meeting, besides several of the papers, will be found elsewhere in the columns of the JOURNAL.

